# Chromatography Based Metabolomics and* In Silico* Screening of* Gymnema sylvestre* Leaf Extract for Its Antidiabetic Potential

**DOI:** 10.1155/2019/7523159

**Published:** 2019-01-06

**Authors:** Shabana Parveen, Mohd Hafizur Rehman Ansari, Rabea Parveen, Washim Khan, Sayeed Ahmad, Syed Akhtar Husain

**Affiliations:** ^1^Human Genetics Laboratory, Department of Bioscience, Jamia Millia Islamia, New Delhi 110025, India; ^2^Bioactive Natural Product Laboratory, School of Pharmaceutical Education and Research, Jamia Hamdard, New Delhi, India

## Abstract

*Gymnema sylvestre*, popularly known as gurmar, is extensively used in traditional systems of medicine for diabetes, stomach ailments, liver diseases, and cardiac disorders. Dried leaf powder of* G. sylvestre* was extracted through soxhlation using 70% (v/v) alcohol. The hydroalcoholic extract was concentrated to 1/4th of its volume and basified to isolate gymnemic acid enriched extract using chloroform. The isolated extract was checked for its antioxidant potential against 1, 1-diphenyl-2-picryl-hydrazyl (DPPH), which showed scavenging activity of 82.31% at 80 *μ*g/mL of extract. Quality control analysis of the extract was carried out by TLC. Chloroform and methanol (9.5:0.5, v/v) were used as a solvent system and separated compounds were detected at 254 and 366 nm. A total of 13 metabolites were separated. However, major peaks were at R_f_ 0.12, 0.69, 0.79, and 0.85. Further, UPLC-MS fingerprinting of the extract was done using acetonitrile and 0.5% formic acid in water as mobile phase in gradient elution mode. A total of 21 metabolites were separated and tentatively identified from the database. Deacyl gymnemic acid and quercetin are the two major metabolites found in the extract. Gymnemic acid, deacyl gymnemic acid, and quercetin were docked with ten different proteins associated with glucose metabolism, transport, and glucose utilization. It has been observed that gymnemic acid was more potent than deacyl gymnemic acid in terms of binding affinity towards proteins and showed a favorable interaction with amino acid residues at the active site. Thus, the present study gives an insight of identified metabolites with protein interaction and a reason for the hypoglycemic potential of deacyl gymnemic acid enriched extract, which can be further explored for* in vitro* and* in vivo* studies to establish its phytopharmacological and therapeutic effect.

## 1. Introduction

Diabetes mellitus (DM) describes a metabolic disorder characterized by a deficiency in insulin production and its action or both [1]. It is thriving distributed in nearly all countries and constantly increases in numbers and implication, as varying quality of life lead to reduced physical activity and increased obesity in populations. That leads to prolonged hyperglycemia with variabilities in most metabolic processes inside the human body [2]. As per global concern World Health Organization (WHO), 347 million people worldwide are suffering from DM, with the estimate that it will be the seventh leading cause of death in 2030. A total of 1.5 million deaths are directly triggered by diabetes in 2012. It was the eighth leading cause of death among both sexes and the fifth leading cause of death in women. When chewed, the fresh leaves of* G. sylvestre* have the outstanding property of paralyzing the sense of sweet taste substance for few times. The gymnemic acid molecules in terms of atomic arrangements are analogous to that of glucose molecules. These types of molecules fill the receptor location on the taste buds thereby stopping its activation by sugar molecules existing in the food. This, up-to-date study showed that the most general medicinal plants with remarkable antidiabetic importance in terms of their mechanism and modes of action together with the methodology part used for their quality, safety, and efficacy assessment to explore the biological standardization of thousands of traditionally used medicinal plants both* in vitro* and* in vivo* metabolomics approach with chromatographic profiling to assess the claimed activity with the aim of finding potent antidiabetic markers from the natural resources [3]. In Indian systems of medicine, i.e., Ayurveda, the* G. sylvestre* prominently used in the therapy of dyspepsia, constipation, and hyperglycemia [4] hemorrhoids, jaundice vesicle, renal calculi, asthma, cardiopathy [5] amenorrhea, bronchitis, and leukoderma [6, 7]. The ethanolic extract of* G. sylvestre* leaves showed the presence of eleven different isoforms of gymnemic acids with different molecular weights (gymnemic acid I to gymnemic acid XI). The major phytoconstituents found in* G. sylvestre* are gymnemic acid (GA), gudmarine, and saponins. Gymnemic acid is a pentacyclic triterpenoid, the main active principle displaying antidiabetic activity [6]. The plant derived extract of* G. sylvestre* has already reported to have direct insulinotropic activities on *β* cells and isolated islets of human* in vitro* [8]. Moreover, antidiabetic potential,* G. sylvestre,* has the capability of total cholesterol and lower triglyceride in serum and its antiatherosclerotic potential were almost similar to that of a standard lipid-lowering agent clofibrate. Some studies reflected the ability of* G. sylvestre* to inhibit the formation of advanced glycation end products and sorbitol accumulation [9].

Due to the presence of specific metabolites, it has been used for different therapeutic purposes. Furthermore, these herbal materials have the significant application for various phytopharmacological applications. Several herbal preparations containing the dried leaves of* G. sylvestre* or its extract are being used for various therapeutic purposes. These plant materials are being used in traditional system of medicine for different disease especially in diabetes. Antidiabetic potential of* G. sylvestre* leaves has been reported but its metabolomic characterization has not been fully explored. Less scientific data are available on the bioactive metabolites responsible for its antidiabetic activity. In our study, we have qualitatively analyzed the number and category of metabolites present in extract through LC-MS and identified the bioactive metabolites through* in silico* screening. Further, we have tested that hydroalcoholic extract has been tested for its antidiabetic potential using* in vitro* and* ex vivo* approaches. In this context our study provides solid scientific evidence in support of its antidiabetic activity. We have authenticated and extracted the leaves of* G. sylvestre.*

## 2. Methodology

### 2.1. Plant Material and Extract Preparation

The leaves of* G. sylvestre* obtained from Botanical Garden of Jamia Hamdard, New Delhi, and authenticated as per the standard protocol specified in Ayurvedic Pharmacopoeia. The authenticated plant materials have been deposited in the Bioactive Natural Product Laboratory for future reference with a voucher specimen number JH/SPER/BNPL/Shabana/2014/GS. The plant sample was washed, shade dried, and coarsely powdered. The powdered drug materials (200 g) of* G. sylvestre* were defatted with petroleum ether and extracted through soxhlation using 70% (v/v) alcohol for 24 h. The hydroalcoholic extract was concentrated to 1/4th of its volume by rotary vaporization under reduced pressure. The extract was filtered and subjected to basify to isolate gymnemic acid enriched extract using chloroform. The extractive value and % yield of extract were calculated and stored at 4°C for bioactivity and quantitative analysis.

### 2.2. Total Phenolic and Flavonoid Content

Through the Folin-Ciocalteu method, the total phenolic content in the hydroalcoholic extract of* G. sylvestre* was determined according to the procedure described in the literature [10]. Different concentrations of gallic acid solutions (as the standard equivalent of phenol) were used for establishing the calibration curve which was further used for the determination of phenol content. All the experiments were carried out in triplicate. The obtained regression equation from the calibration plot was used for the determination of total phenolic content and expressed as mg of gallic acid equivalent per gram of extract. Aluminum chloride (AlCl_3_) colorimetric method was used for the determination of total flavonoid content [10]. The total flavonoid content in the hydroalcoholic extract was calculated from a calibration curve of standard (rutin) by using its different dilutions concentrations ranging from 10 to 100 *μ*g/mL. The total flavonoid content was expressed as mg/g of rutin equivalent.

### 2.3. Determination of Antioxidant Potential

The antioxidant potential of the extract was determined by 1, 1-diphenyl-2-picryl-hydrazyl (DPPH) assay. The stock solution of different concentrations of extract (10, 20, and 40 *μ*g/mL) was mixed with 1.0 mL of a methanolic solution of DPPH (1.0 *μ*g/mL) and incubated for 30 min in dark at room temperature. Then, the absorbance was recorded at 517 nm using a UV-visible spectrophotometer. Trolox was used as standards for comparison.

### 2.4. In Vitro Carbohydrate Digesting Enzyme Inhibition Assay

For the determination of the *α*-amylase inhibitory potential of extract, previously developed method was followed [10]. Briefly, accurately weighed 5 mg of enzyme was dissolved in 10 mL of 20 mM phosphate buffer (pH 6.9) at 37°C, while the extract was dissolved in dimethylsulfoxide and diluted in phosphate buffer. Different concentration of extract ranging from 100 to 1000 *μ*g/mL was used for the determination of *α*-amylase inhibition potential. One mL of diluted extract and 1.0 mL of enzyme solutions (0.5 mg/mL) were mixed together and incubated at room temperature for 30 min. After the completion of incubation, 1.0 mL of 0.5% (w/v) starch solution was added to the mixture and then again kept for 10 min at room temperature. About 2.0 mL of dinitrosalicylic acid was added to the reaction mixture and heated in boiling water for 5 min to stop the previously ongoing reaction. The resulting mixture was cooled and absorbance was measured calorimetrically at 565 nm.

For the determination of *α*-glucosidase inhibitory potential of extract, previously reported procedure [10] was followed. Extract prepared for *α*-amylase assay was used for *α*-glucosidase assay also. While an enzyme solution (1.0 U/mL) was prepared in 10 mM phosphate buffer (pH 6.8). Briefly, the 100 *μ*L of diluted extract and 200 *μ*L of enzyme solution were incubated at 37°C for ten minutes. Then, 100 *μ*L of p-nitrophenyl-*α*-D glucopyranoside (PNPG) solution (5.0 mM) in 10 mM phosphate buffer (pH 6.8) were added to start the reaction and the mixture was incubated at 37°C for 30 min. Then the reaction was stopped after the addition of 2.0 mL of 0.1 M sodium carbonate (Na_2_CO_3_). Finally, the absorbance was recorded at 405 nm of the yellow colored p-nitrophenol freely released from p-nitrophenyl-*α*-D-glucopyranoside. Acarbose was used as the positive control and the results were expressed as the inhibition rate (%) of enzymatic activity and figured by the beneath equation:

(1) %  Inhibition  of  enzyme  activity=Abscontrol−Abssample×100Abscontrol

where Abs_control_ is the OD of reaction without extract or standard and Abs_sample_ is the OD of the reaction of with extract or standard.

### 2.5. Ex Vivo Glucose Uptake

The inhibition assay of intestinal glucose uptake was determined in rat hemidiaphragm. About overnight fasted rats were used for this assay. Animals were euthanized by anesthesia and dissected to isolate diaphragm. It was immediately dipped in the ice-cold Krebs-Henseleit buffer which was previously equilibrated with 95% oxygen, 5% carbon dioxide in a cylindrical vessel of organ bath. The extract was added in the same compartment and incubated at room temperature for one hour. Further, glucose solution was added and again incubated for 30 min at room temperature. After the completion of incubation, a sample from the supernatant was collected and the unabsorbed glucose was estimated using glucose estimation kit which was commercially utilized. Extracts with different concentrations ranging from 25 to 100 *μ*g/mL were used. Inhibition of glucose was measured by the following formula:

(2) $$\begin{array}{c} \text{Inhibition of glucose}=\frac{\left(C2-C1\right)}{g\text{ of hemidiaphragm}} \end{array}$$

where C2 and C1 were final and initial glucose concentration after the incubation.

Skeletal muscle was isolated from the dissected animals and used for glucose uptake assay. The previously standardized protocol was used to check the effect of the extract on glucose uptake in isolated rat skeletal muscle [11]. It was immediately dipped in ice-cold Kreb's buffer which was previously equilibrated with 95% oxygen and 5% carbon dioxide in a cylindrical vessel of organ bath. The muscle was with buffer for three times. The extract was added in the same compartment and incubated at room temperature for one hour. Further, glucose solution (20 mM) was added and again incubated for 30 min at room temperature. In control reaction, only glucose was added while, in case of test reaction, extract with different concentration (25-100 *μ*g/mL) was used separately. Metformin standard was used as positive control. Before and after the completion of incubation, 1.0 mL of supernatant was collected and the unabsorbed glucose was estimated using glucose estimation kit which was commercially utilized. The amount of glucose uptake by per gram of muscle was measured by the following formula:

(3) $$\begin{array}{c} \text{Muscle glucose uptake}=\frac{\left(C1-C2\right)}{g\text{ of muscle tissue}} \end{array}$$

where C1 and C2 are the concentrations of glucose before and after incubation, respectively.

### 2.6. Effect of Extract on Glucose Uptake in Yeast Cells

Commercially utilized baker's yeast (*Saccharomyces cerevisiae*) was obtained from Institute of Microbial Technology, Chandigarh, India, and used for this assay. The obtained microbes were subcultured in potato dextrose agar medium and further suspension culture was prepared in potato dextrose broth. The culture medium was centrifuged at 3,000×g for 5 min and the cell pellet was washed in distilled water until the supernatant fluids were clear. About 10% v/v of cell pellet suspension was prepared with the supernatant fluid. One mL of the glucose solution was added with 1 mL of extract and incubated at 37°C for 10 min. The reaction was started by adding 100 *μ*L of yeast suspension to the above mixture. The resulting reaction mixture was vortexed and incubated at 37°C for 60 min. After the completion of the mixture, it was centrifuged and the glucose concentration was measured from the supernatant [10]. Different concentrations of glucose (5, 10, and 25 mM) and extract (250, 500, 750, and 1000 *μ*g) were tested. The percentage increase of glucose in yeast cell was determined using the following equation:

(4) Percentage  increase  in  glucose  uptake=Abssample−Abscontrol×100Abssample

where Abs_control_ is the reaction without extract and Abs_sample_ is the reaction with extract.

### 2.7. TLC Fingerprinting of Extract

The hydroalcoholic extract was dissolved in HPLC grade methanol and filtered through 0.25 *μ*M membrane filter. The chromatography analysis was performed on aluminum TLC plates coated with 0.2 *μ*M layers of silica gel 60F_254_ (Merck Millipore, Germany). Samples were applied with 4.0 mm wide band and 8.3 mm gap between each band by the use of a Linomat V sample applicator (CAMAG, Switzerland). The sample concentration was 10 mg/Ml and 5.0 *μ*L sample was applied with a constant sample application rate of 5.0 *μ*L. For the best separation of metabolites, chloroform: methanol (95:05, %v/v) was used as a mobile phase and the plate was developed in a 20 × 10 cm twin-trough glass chamber with linear ascending mode up to 80 mm. Further, the plate was removed from chamber and air dried. The developed plate was scanned at two different wavelengths, i.e., 254 and 366 nm with a TLC scanner III (CAMAG, Switzerland) with slit dimension of 4.0 × 0.30 mm, and the scanning speed was 10 mm/s. The sample application and scanning were operated by winCats software. The developed method was validated as per the ICH guidelines for quality control of herbal drugs and botanicals [12, 13]. The peak areas of triplicate samples were used for analyzing the metabolic diversity of hydroalcoholic extract.

### 2.8. Ultraperformance Liquid Chromatography-Mass Spectrometry Analysis of the Extract

Water's ACQUITY UPLC™ system (Serial No. #F09 UPB 920M; Model code # UPB; Waters Corp., MA, USA) equipped with a binary solvent delivery system, column manager, an auto sampler, and a tunable MS detector (Serial No # JAA 272; Synapt; Waters, Manchester, UK) was used for UPLC-MS analysis of extract. The extract was chromatographically separated in previously degassed mobile phase consisting of 0.5% v/v formic acid in water (A) and acetonitrile (B) in gradient elution mode (initially 100% A and hold for 5 min; further, decreased to 5% A in 20 min). Water's ACQUITY UPLC™ BEH C18 (100.0 × 2.1 mm × 1.7 *μ*m) column was used and flow rate of mobile phase was 0.4 mL/min. The column manager and sample manager temperature were set to 35 ± 2°C and 25 ± 2°C, respectively. About 10 *μ*L of sample was injected with the split mode of 5:1 with the help of autoinjector and the pressure of the system was set to 15000 psi.

The separated metabolites were detected by MS detector on a quadruple orthogonal acceleration time of flight tandem mass spectrometer (Waters Q-TOF Premier TM). The nebulizer gas and cone gas were set to 500 L/h and 50 L/h, respectively. The source temperature of MS detector was set to 100°C. The capillary voltage and cone voltage were set to 3.0 kV and 40 kV, respectively. For collision of ion, argon gas was used at a pressure of 5.3 × 10^−5^ Torr. The Q-TOF Premier™ was operated in scan mode with resolution over 8500 mass with 1.0 min scan time and 0.02 s interscan delay. Both UPLC and the mass detector were operated by using Mass Lynx V 4.1 software incorporated with the instrument. The separated compounds were identified based on their m/z value through literature survey [10].

### 2.9. In Silico Screening

To understand the binding mechanisms of active constituents of gymnema leaves, molecular modelling studies were accomplished for deaclgymnemic acid, gymnemic acid, quercetin, and the aglycone moiety gymnemagenin with target proteins by the mopac 6 software package (Stewart Computational Chemistry, Colorado Springs, USA). Different proteins were presumed to interact with targeted molecules (deaclgymnemic acid, gymnemic acid, quercetin, and gymnemagenin). Nine different proteins such as (dipeptidyl peptidase, glucokinase, glutamine fructose-6-phosphate amidotransferase, AMP kinase, GLUT-2, stearoyl-coA desaturase, GLUT-4, sulfonylurea receptor, and mitochondrial Na+/K+ exchanger) were selected for docking analysis. All the docking calculations were achieved on different protein models. In AutoDock tools, solvation parameters, essential hydrogen atoms, and Kollman united atom type charges were added. Autogrid program was employed for generation of affinity (grid) maps of × Å grid points and 0.375 Å spacing. Van der Waals and the electrostatic terms were generated by AutoDock parameter set and distance dependent dielectric functions, respectively. Simulations of docking were executed using the Lamarckian genetic algorithm and the Solis and Wets local search method. Initial position, orientation, and torsions of the ligand molecules have been randomly selected. During docking, all rotatable torsions were released. A translational step of 0.2 Å was used, whereas 5 quaternion and torsion steps were utilized in each search. In each docking experiment, two different runs were set and it was terminated after the assessment of maximum 250000 energy was reached. The structure of molecules in mol format was generated in the CDX format using the tool ChemDraw Ultra 7.0.1 (CambridgeSoft Corporation, Cambridge, USA) and transformed to input ligand format (pdb) for docking by OpenBabel version 2.3.2 Open Babel: An open chemical toolbox (Journal of Cheminformatics 2011, 3:33).

## 3. Results

The dried leaves of* G. sylvestre *were defatted through pet ether (60-80°C). The hydroalcoholic extract (70%) was prepared from soxhlation process. The percentage yield of 70% hydroalcoholic extract was found to be 24.30% w/w. The extract was filtered and basified to isolate the gymnemic acid enriched fraction by successive solvent selection process using chloroform with 1.31% w/w yield. Further, the extract was dried and stored at 4°C until use.

### 3.1. Phenolic and Flavonoid Content of Extract

The total phenolic and flavonoid content of the hydroalcoholic extract was determined from the calibration curve of gallic acid (r^2^ = 0.998) and rutin (r^2^ = 0.989), respectively. The total phenolic content was found to be 29.36 mg of gallic acid equivalents per gram of extract, while flavonoid content was 18.65 mg of rutin equivalents per gram of extract. This extract is enriched with phenols and especially flavonoids which are mainly responsible for the antioxidant potential of extract. The antioxidant activity was due to the presence of free hydroxyl group present in flavonoids of extract. Free hydroxyl group scavenges the free radicals caused better antioxidant potential. The antioxidant activity of flavonoids, which include flavones, flavanols, and condensed tannins, depends on the presence of free (OH) hydroxyl groups, especially 3-OH, since the present report of antioxidant activity of hydroalcoholic extract suggesting a complete profiling via phytochemical and metabolomics profiling needs to be done to identify the other active phenolic and flavonoid components in the field of drug discovery and development.

### 3.2. Antioxidant Potential

Due to the simplicity in a biochemical reaction, the free radical scavenging activity of any plant extract is commonly used to determine by DPPH radical. Hydroalcoholic extracts are the rich sources for phenols and flavonoids, which are having redox properties with antioxidant potential. Our phytochemical screening revealed that it has the major abundance of phenolic and flavonoid metabolites. The hydroalcoholic extract had clearly shown the strong antioxidant potential against all free radicals. The results clearly showed that DPPH scavenging activity was 14.84 ± 0.12, 21.14 ± 0.20, and 34.36 ± 0.45 of mMTR equivalent, at a concentration of 10, 20, and 40 *μ*g/mL of* G. Sylvestre extract*, respectively, while that of the control, i.e., ascorbic acid was 41.36 mMTR when the concentration was 35 *μ*g/mL. The antioxidant potential which was equivalent to DPPH scavenging was increased with respect to the extract concentration and it was increased to a concentration of 40 *μ*g/mL. Beyond the level of extract used, no increment in antioxidant potential was observed. The hydroalcoholic extract enriched with flavonoids which have the potential to scavenge the free radicals associated with different ailments and biological cycle, singlet oxygen, and other oxidizing molecules. Apart from the scavenging the free radicals, flavonoids suppress the production of reactive oxygen species, quenched the trace elements, and upregulate antioxidant defenses which are directly involved in the production of free radical. Similar actions were also reported in extract enriched with phenolic content.

### 3.3. Carbohydrate Digesting Enzyme Inhibition Potential

**Figure 1:**
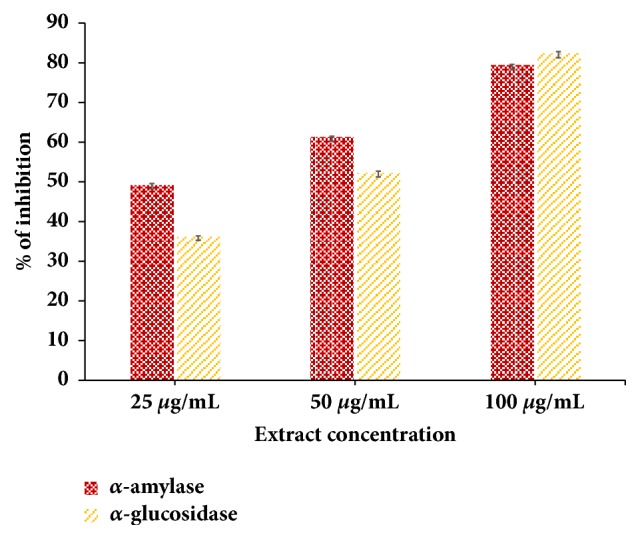
Carbohydrate digesting enzyme (*α*-amylase and *α*-glucosidase) inhibition potential of* G. sylvestre* extract.

The different concentration of 25 *μ*g/mL, 50 *μ*g/mL, and 100 *μ*g/mL of hydroalcoholic extract clearly showed that the % inhibition of *α*-amylase and *α*-glucosidase activity consistently increases with concentration-dependent manner, respectively. The hydroalcoholic extract has clearly shown the remarkable potential of carbohydrate-digesting enzyme at a specific concentration. Carbohydrate digesting enzyme (*α*-glucosidase) inhibitory potential significantly increased with increase in the concentration of hydroalcoholic extract. The carbohydrate enzyme inhibition potential of extract has been shown in Figure 1.

### 3.4. Yeast Cell Uptake

**Figure 2:**
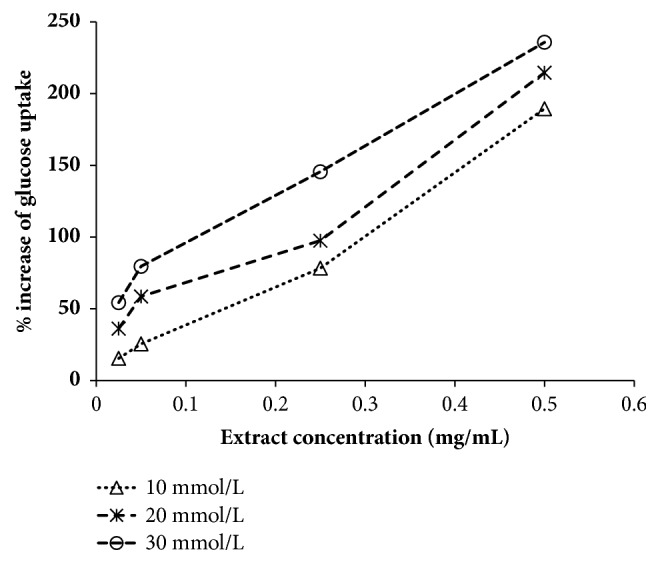
Effect of extract on glucose uptake in yeast cell uptake.

By facilitated diffusion process in baker's yeast follows glucose transport process. The process in which glucose was uptaken by skeletal muscle was similar to the process glucose transport in a yeast cell. After specific incubation time period in experimental medium, the amount of the glucose remained in the process of glucose uptake by the yeast cell. It consistently increased in a dose-dependent manner.  Figure 2 clearly shows that the percent increases in glucose uptake in yeast cells at different glucose concentrations, i.e., 10 mmol/L, 20 mmol/L, and 30 mmol/L with respect to concentrations of extract. A concentration-dependent glucose uptake was increased in a yeast cell in the presence of extract. In case of positive control, metformin was used and it also showed the increased glucose uptake in yeast cell. However, hydroalcoholic extract showed greater effectiveness in glucose uptake by yeast cells as compared to positive control. Glucose uptake in yeast cell followed diffusion process which was similar to glucose uptake in skeletal muscle. Thus, these results indicate that hydroalcoholic extract will increase the glucose uptake in skeletal muscle or it will cause an increment in peripheral glucose utilization, while a significant difference in glucose uptake in yeast cell was observed when it was incubated with extract as compared to yeast cell incubated with metformin.

### 3.5. Ex Vivo Antidiabetic Potential

**Figure 3:**
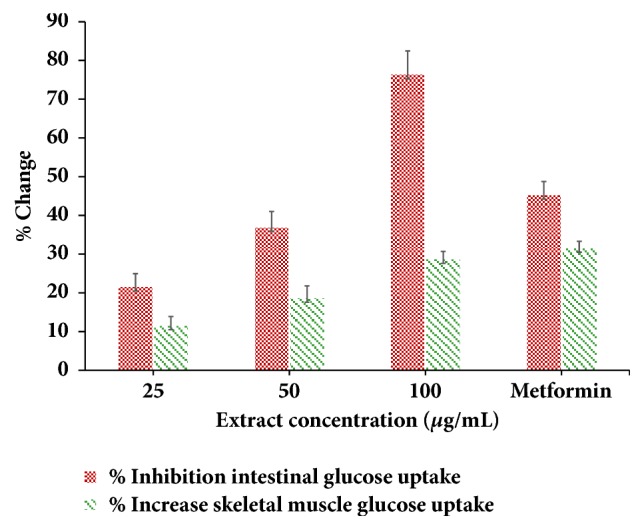
Effect of extract on intestinal absorption inhibition and percentage glucose uptake in skeletal muscle.

Generally, researchers have used the isolated diaphragm to check the effect of metabolites on the intestinal glucose inhibition. In our study, we have checked the effect of hydroalcoholic extract of* G. sylvestre* leaves on intestinal glucose absorption. A marked decrease in glucose absorption in the intestine by extract was recorded as compared to control. A dose-dependent glucose absorption inhibition was recorded (Figure 3). A maximum glucose absorption inhibition (76.25%) has been observed at a concentration of 100 *μ*g/mL of extract. Similar results were obtained in glucose uptake assay in skeletal muscle. The effects of the extract on glucose uptake in isolated rat skeletal muscle are shown in Figure 3. A maximum 28.6% glucose uptake was increased at a dose of 100 *μ*g/mL of extract as compared to control.

### 3.6. TLC Fingerprinting Analysis

**Figure 4:**
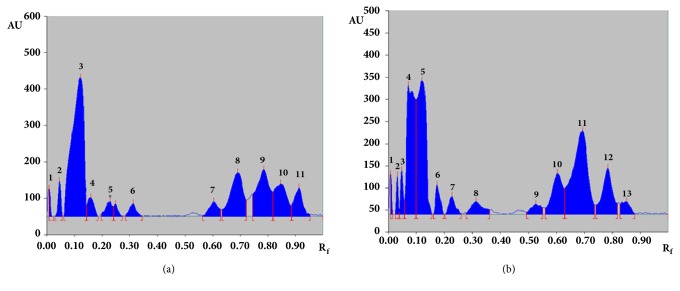
TLC chromatogram of extract scanned at (a) 254 nm and (b) 366 nm.

For TLC fingerprinting analysis, hydroalcoholic extract was dissolved in methanol, filtered, and analyzed through 0.25 *μ*M membrane filter. Chromatographic separation was performed by using chloroform: methanol: formic acid, 9:5:0.5, v/v/v, as the mobile phase. The developed plate was scanned at two different wavelengths, 254 nm and 366 nm (Figure 4). The TLC analysis of hydroalcoholic extract clearly showed the separation of total 15 metabolites. Scanning at 254 and 366 nm, a number of compounds analyzed are 12 and 13, respectively (Table 1). However, major components in terms of peaks area in chromatogram were found at R_f_ 0.12 (40.3%), 0.69 (13.7%), 0.79 (14.72%), 0.85 (9.99%) and 0.91 (5.94%) at 254 nm. While at 366 nm, major compounds found at R_f_ 0.07 (20.23%), 0.12 (22.51%), 0.60 (8.53%), 0.69 (23.62%), and 0.79 (8.79%). One major peak was found at R_f_ 0.07 (20.23%) while scanning at 366 nm but this peak was not found at 254 nm. Similarly, one peak at R_f_ 0.91 (5.94%) was found when scanned at 254 nm but found absent at 366 nm.

### 3.7. UPLC-MS Analysis

**Figure 5:**
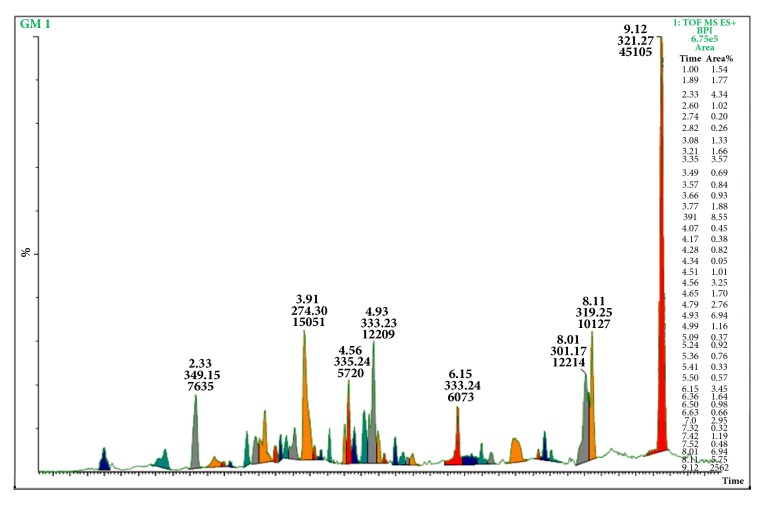
UPLC-MS chromatogram of extract.

**Figure 6:**
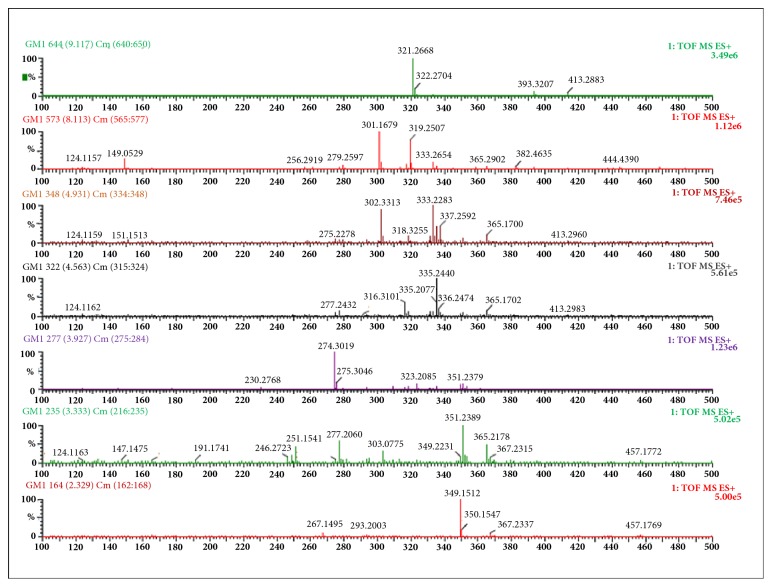
Mass spectrum of major abundant metabolites analyzed through UPLC-MS.

All the metabolites present in the hydroalcoholic extract were dissolved in methanol and, based on this assumption, the methanolic solution was analyzed through UPLC-MS for their complete metabolic profiling and identification of diversity of metabolites.  Table 2 summarizes all the metabolites characterized in* G. sylvestre* extract eluted at different retention times, experimental m/z, and tentative name with nature of compounds. A total of 58 most abundant metabolites were analyzed and identified through m/z value and from literature survey. By comparing chromatogram of blank with the chromatogram of extract, a clear and contrast chromatogram was observed based on their retention time (Figure 5). LC-MS fingerprinting of the* G. sylvestre* extract was done using 0.5% v/v formic acid in water (A) and acetonitrile (B) as mobile phase in gradient elution mode. A total of 58 metabolites were separated and tentatively identified from the database. Deacyl gymnemic acid and quercetin were the two major metabolites found in the extract with m/z value of quercetin, conderitol, deacylgymnemic acid, niacin, ascorbic acid, rutin, kaempferol, niacin, D-quercetin, and stigmasterol. Mass spectra of major metabolites have been shown in Figure 6.

**Figure 7:**
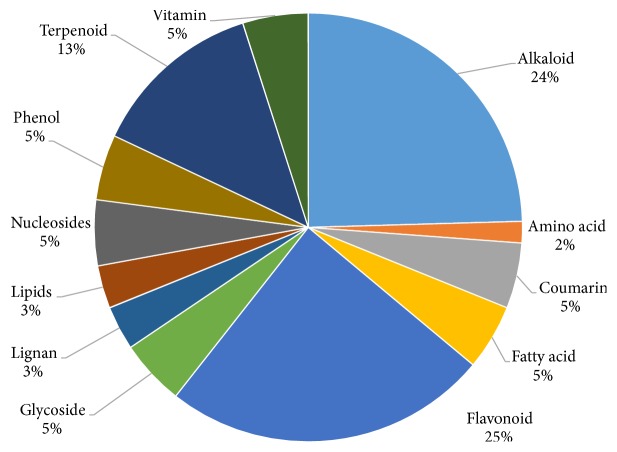
Categorization of analysed metabolites.

Major groups of tentatively identified metabolites are alkaloids (25.8%), amino acids (1.7%), coumarins (5.2%), fatty acids (5.2%), flavonoids (25.8%), glycosides (5.2%), lignans (3.5%), lipids (3.4%), nucleosides (5.2%), phenols (5.2%), terpenoids (13.7%), and vitamins (5.2%) (Figure 7). Our investigations revealed that the hydroalcoholic composed of major phenolic and flavonoid metabolites which are responsible for its therapeutic potential. Thus, for the analysis of low and high abundant metabolites with wide polarity range, UPLC-MS method seems to be the best method of analysis.

### 3.8. In Silico Screening

**Figure 8:**
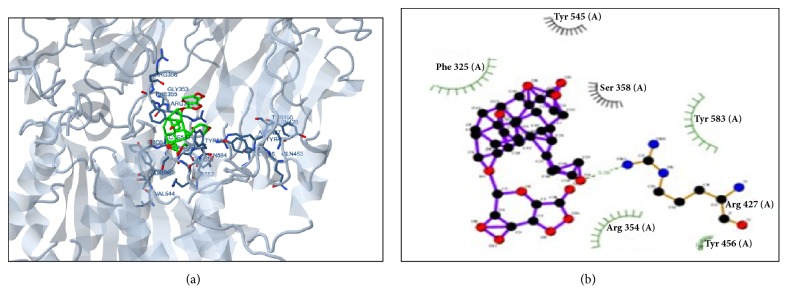
(a) 3D and (b) 2D interaction of deacyl gymnemic acid with DPP4.

Gymnemic acid, deacyl gymnemic acid, and quercetin were docked with ten different proteins associated with glucose metabolism, transport, and glucose utilization. The docking of tested metabolites with targeted proteins was therefore performed, and corresponding fitness scores were determined. High fitness scored metabolites were subjected to the elucidation of their interaction surface and total intermolecular energy with targeted molecules and proteins separately [14]. Four ligands (deacyl gymnemic acid, gymnemagenin, gymnemic acid, and quercetin) were selected for this study. All these metabolites showed good interaction with glucose transporter and deacylgymnemic acid showed better affinity as compared to other metabolites. Almost all the compounds showed good affinity and the estimated free energy of ligand-protein interaction was found < -5.0 for the receptor proteins in this interaction indicating that affinity of these proteins towards targeted metabolites might be changed after oral administration of extract. It has been observed that gymnemic acid was more potent than deacyl gymnemic acid in terms of binding affinity towards proteins and showed a favorable interaction with amino acid residues at the active site. Docking summary of metabolites docked with proteins associated with diabetes has been shown in Table 3. Some of the major protein interactions are shown in Figure 8. Thus, the present* in silico* screening study gives an insight of tentatively identified metabolites with a specific protein. The data obtained from* in silico* screening will help to define/predict the mechanism behind the antidiabetic potential of metabolites present in the extract of* G. sylvestre* through ligand-receptor interaction based on binding energies or fitness score [15]. Further, a molecular-based study is required for confirmation of the above-proposed mechanism.

## 4. Discussion

The use of medicinal plants and traditional medicine in developed and developing countries is becoming popular as a medical alternative in the treatment of various ailments including diabetes. It has been known that oxidative damage is associated with a number of disease processes and possible mechanism including diabetes mellitus. In the present study, the hydroalcoholic extract from* G. sylvestre *leaf exhibited potent antioxidant activities and increased the activity of enzymes beneficial in the prevention of diabetes.

From ethnopharmacological relevance point of view, the extract of* G. sylvestre* has been used for therapeutic purposes and current scientific data are available for its several other therapeutic actions such as antioxidant, anticancer, anti-inflammatory, antidiabetic, hypolipidemic, and hypotensive [7]. Our study clearly demonstrated that the hydroalcoholic extract of* G. sylvestre* was utilized with a well-defined low dose as mentioned in Ayurved Pharmacopeia for its antidiabetic potential.

We have obtained more yield of hydroalcoholic extract as compared by using general extraction procedure through maceration with water yielding and this can be used in industrial scale also. The total amount of phenolic and flavonoid content in the extracts from* G. sylvestre* leaf was determined. Gallic acid and rutin were used to express the phenolic and flavonoid content, respectively. The content of phenolic and flavonoids found in natural plants is known to have a number of beneficial health effects associated with natural antioxidants suppressing the LDL cholesterol oxidation [16] and decreasing the risk of heart disorder [17]. The hydroalcoholic extract showed potent antioxidant potential and is expressed as Trolox equivalent. The metabolites present in the extract were able to scavenge the DPPH and we obtained the similar type of activities as ascorbic acid showed. The results obtained from antioxidant assay indicate that hydroalcoholic extract of* G. sylvestre* leaf contains potent antioxidants potential. It has earlier been proved that plants rich in polyphenolic compounds, such as phenolic acids and flavonoids, possess outstanding antioxidant activities [18].

The results clearly showed that the in vitro *α*-amylase and *α* glucosidase inhibition test displayed that the hydroalcoholic extract of* G. sylvestre *had a potent inhibitory effect and it showed better activity as compared to acarbose as a standard drug. This in vitro finding suggested that the hydroalcoholic extract of* G. sylvestre* can be able to significantly reduce the postprandial level by inhibiting the activity of *α*-amylase and *α*-glucosidase, which are important enzymes in the digestion of the complex carbohydrates into absorbable monosaccharides in the food. Glucose uptake in yeast cell follows the passive diffusion as similar to glucose uptake in human skeletal muscle [19]. If any extract is able to increase glucose uptake in yeast cell which will also be able to increase glucose uptake in skeletal muscle [20]. The hydroalcoholic extract was able to increase the glucose uptake in yeast cell more than two hundred percent as compared to control condition without having extract (Figure 2). Thus this yeast cell uptake is an evidence of the antidiabetic potential of hydroalcoholic extract. Glucose uptake in yeast cell has been done very first time for* G. sylvestre* extract. This result was further supported by* ex vivo* glucose uptake assay. The hydroalcoholic extract was able to increase the glucose uptake in skeletal muscle isolated from rats. In the presence of extract, a significant increase in glucose uptake was observed as compared to normal conditions (Figure 3). On the other hand, we have checked the effect of the hydroalcoholic extract on glucose absorption inhibition in intestine. We found that the extract was able to inhibit the glucose absorption in intestine. From both the ex vivo experiments suggest that the extract is able to increase the peripheral glucose utilization and also decrease the glucose absorption. Thus, it will be more powerful antidiabetic medicine for the patients who are suffering from type 2 diabetes [21]. This* ex vivo* study provided the biological environment and gives the possible mechanism of the hydroalcoholic extract of* G. sylvestre* for its antidiabetic potential.

Quality control analysis is one of the major concerns for herbal formulation. TLC fingerprinting is usually used to get the metabolite patterns of any extract so that we can identify the extract in future. If any extract will have the same TLC pattern, it must show the same biological activity and we know its TLC fingerprint. In this context, we have developed the TLC method and analyzed our extract. The developed method was reproducible and we have total 13 metabolites present in the hydroalcoholic extract (Figure 4). This TLC fingerprint can be used for its quality control analysis and regulatory bodies to assure its quality and safety [12]. Further, to identify the different metabolites present in the extract, we performed UPLC-MS analysis. UPLC-MS is the most powerful tool for the identification of polar and nonpolar metabolites. In our experiment, we chromatographically separated and tentatively identified based on their mass. Total 58 metabolites were analyzed and identified through UPLC-MS (Table 2). Further, identified metabolites were categorized (Figure 7). HPTLC fingerprinting and LC-MS analysis identify the constituents present in the hydroalcoholic extract which usually polar secondary metabolites such as glycosides, phenols, nucleosides, terpenoids, vitamins, lipids lignans, fatty acids, saponin, and tannins and some primary metabolites such as glycosides, vitamins, and proteins. Metabolomics is a useful and powerful tool for the chemical and pharmacological standardization of plant extract [22] and it has the potential to make a revolution in research of natural product and to advance the scientifically development of herbal based medicine. Metabolomes of some important medicinal plants are particularly a valuable natural resource for the evidence-based development of new nutraceuticals and phytotherapeuticals. This is the first time we identified the metabolites present in hydroalcoholic extract which were further categorized. This study will be helpful for the researchers who are working in* G. sylvestre*.

In order to predict the mechanism, we have checked the affinity of major metabolites present in the extract with the proteins associated with glucose biosynthesis, metabolism, and utilization. We have selected total four major metabolites present in extract and identified through UPLC-MS. These metabolites were docked with protein and we have analyzed their affinity (Table 3). It has found that gymnemic acid is more potent than deacyl gymnemic acid in terms of affinity towards selected protein. It was observed that almost all compounds are with low binding energies and clearly shows that every compound for the enzyme was found with good affinity.* In silico* study of responsible metabolites gives an insight of tentatively identified metabolites with a specific protein. The* in silico* approach can be used to model the interaction and binding energy between a small molecule and protein at the atomic level, which allows us to characterize the behavior of metabolites in the binding site of target proteins as well as elucidate the preliminary mechanism of action of molecules.

In summary, we chromatographically characterized the hydroalcoholic extract of* G. sylvestre* leaves extract and we have tested its antidiabetic potential by using* in vitro*,* ex vivo*,* in silico,* and metabolomics approaches. The results of the study will be helpful for the development of phytopharmaceuticals which can be used for the management of diabetes.

## 5. Conclusion

The present study substantiated the hypoglycemic potential of* G. sylvestre* leaves, which has been used since long for the management of diabetes. The hydroalcoholic extract exhibited the hypoglycemic activity by increasing the glucose uptake in skeletal muscle, inhibiting intestinal glucose absorption, and by scavenging redox molecule.* In silico* study predicted the mechanism behind the antidiabetic potential of extract. However, a molecular level study is needed to be performed for better clarification and providing more scientific data. Thus, hydroalcoholic extract enriched with gymnemic acid and deacylgymnemic acid can be explored for the development of phytopharmaceuticals.

## Figures and Tables

**Table 1:** TLC fingerprinting of hydroalcoholic extract of *G. sylvestre* leaves.

**S. No.**	**R**_**f**_	**Area**%	
		**At 254 nm**	**At 366 nm**
1.	0.01	0.98	1.10
2.	0.03	-	1.30
3.	0.05	2.20	1.96
4.	0.07	-	**20.23**
5.	0.12	40.31	22.51
6.	0.16	3.00	2.54
7.	0.23	2.49	2.22
8.	0.25	1.25	-
9.	0.31	2.14	2.98
10.	0.53	-	1.97
11.	0.60	3.28	8.53
12.	0.69	13.70	23.62
13.	0.79	14.72	8.79
14.	0.85	9.99	2.25
15.	0.91	5.94	-
**Total**	**15**	**12**	**13**

**Table 2:** UPLC-MS fingerprinting profile of hydroalcoholic extract of *G. sylvestre*.

**Metabolites**	**m/z**	**Name**	**Class of compound**	**Reference**
M1	350.15	Andrographolide	Labdane triterpenoid	BML80745
M2	367.23	Curcumin	Flavonoid	TY000081
M3	457.17	(-)-Epigallocatechin gallate	Polyphenol cds	TY000083
M4	293.2	Gingerol	Terpenoids	CO000211
M5	267.14	3-Hydroxy-3′-methoxyflavone	Flavonoids	BML80385
M6	351.23	Ajmalicine	Indole alkaloids	FIO00001
M7	277.2	Ascorbic acid magnesium phosphate	Vitamin	PubChem CID:101614363
M8	147.14	Conduritol	Polyphenol cds	PubChem CID: 136345
M9	365.21	Isopentenyl-Adenine-7-glucoside	Pyridine alkaloids	CE000239
M10	251.15	(3aR)-(+)-Sclareolide	Terpenoids	BML80075
M11	303.07	Dihydroquercetin	Flavonoids	BML81120
M12	349.22	Strychnine_N_Oxide	Labdane diterpenoid	CO000416
M13	275.3	Eserine		KO008958
M14	323.2	Quinine	Quinidine alkaloid	BML82035
M15	230.27	7-Diethylamino-4-methylcoumarin	Coumarin	SM884302
M16	335.24	Berberine	Isoquinoline alkaloids	KO008886
M17	316.31	Capillarisin	Sesquiterpnoids	TY000038
M18	336.24	Lobeline	Piperidine alkaloid	BML81620
M19	365.17	Isopentenyl-Adenine-7-glucoside	Pyridine alkaloids	CE000239
M20	413.29	S,R-Noscapine	Benzylisoquinoline alkaloid	CE000163
M21	291.18	Karanjin	Steroidal alkaloid	BML81520
M22	277.24	Linolenic acid	Steroidal alkaloid	BML81605
M23	333.22	Strychnine	Labdane diterpenoid	WA000648
M24	302.33	Hesperetin	Flavonoids	BML81380
M25	337.25	8-Geranyloxy psoralen	Furanocoumarin	BML80640
M26	318.32	Myricetin	Flavonoids	TY000149
M27	275.22	Eserine		KO008958
M28	301.16	Hematoxylin	Neoflavonoids	BML81375
M29	319.25	Coptisine	Benzo[c]phenanthridine alkaloids	TY000106
M30	149.05	Methionine	Sulphur containing amino acid	CE000452
M31	382.46	Dihydrozeatin-9-beta-D-glucoside	Purine nucleosidee	PR020117
M32	279.25	Linoleic acid	Unsaturated fatty acids	EQ331601
M33	365.29	Isopentenyl-Adenine-7-glucoside-[d6]	Pyridine alkaloids	CE000594
M34	444.43	Bufotalin (Saponin)	Saponin	TY000016
M35	124.12	Orcinol	Flavonoids	BML81850
M36	151.15	Cathine	Phenylpropanes	EQ333501
M37	165.14	D-Quercitol	Flavonoids	PubChem CID: 441437
M38	177.09	Ascorbic acid	Vitamin	PubChem CID: 54670067
M39	207.16	Anthraquinone		PubChem CID: 6780
M40	251.16	7-Hydroxy-3-methylflavone	Flavonoids	BML80610
M41	274.31	Phloretin	Dihydrochalchone (Phenol class)	TY000158
M42	301.18	Quercetin	Flavonoids	CE000168
M43	335.25	Senecionine	Pyrrolidizine alkaloids	FIO00235
M44	351.25	4-Methylumbelliferylglucuronide	Benzopyran alkaloids	CE000020
M45	353.26	Chelidonine	Phenylisoquinoline alkaloids	CE000133
M46	353.3	Asarinin	Benzofuran type lignan	BML80780
M47	413.28	Stigmasterol	Benzofuran type lignan	PubChem CID: 5280794
M48	429.4	Ononin	Isoflavonoid	PR020043
M49	523.39	1-Stearoylglycerophosphocholine	Glycerophospholipids	MT000126
M50	549.44	Quercetin-3-(6′′-malonyl)-glucoside	Flavonoid	PR101032
M51	579.45	Naringin	Flavonoid	CE000186
M52	593.3	Kaempferol-3-O-*β*-glucopyranosyl-7-O-*α*-rhamnopyranoside	Glycerophospholipids	PR101010
M53	639.34	Demethoxycentaureidin 7-O-rutinoside	Flavonoids	BML81075
M54	682.4	Deacyl gymnemic acid II	Triterpenoidal saponins	PubChem CID: 44144284
M55	693.52	Rutin 3′′-malonate	Flavonoids	PubChem CID: 10556617
M56	763.56	Gymnemic acid IV	Triterpenoidal saponins	PubChem CID: 14264063
M57	835.59	Triacylglycerol 16:0-16:0-18:0	Unsaturated fatty acids	UT000540
M58	877.51	Triacylglycerol 18:2-18:2-18:2	Unsaturated fatty acids	UT000521

**Table 3:** Docking summary of major abundant analysed metabolites with different proteins associated with diabetes.

**Major metabolites**	**Parameters**	**P-1**	**P-2**	**P-3**	**P-4**	**P-5**	**P-6**	**P-7**	**P-8**	**P-9**
**Deacylgymnemic acid**	Free energy (kcal/mol)	**-6.53**	59.4	-13.17	-9.22	27.07	-8.83	-11.08	-5.57	31.67
	Interact Surface	599.5	614.3	963.6	859.5	778.9	1079.2	1032.1	669.8	698.1
	Intermolecular Energy (kcal/mol)	-7.94	59.1	-13.97	-10.36	26.35	-8.94	-11.77	-6.96	19.28

**Gymnemagenin**	Free energy (kcal/mol)	-8.49	-2.56	59.4	-10.85	19.98	-8.91	-10.81	-7.59	-8.39
	Interact Surface	593.8	646.1	614.3	733.8	481.6	626.8	689.5	558.3	501.2
	Intermolecular Energy (kcal/mol)	-8.79	-2.86	59.1	-11.15	+19.69	-9.21	-11.11	-7.89	-8.69

**Gymnemic acid**	Free energy (kcal/mol)	-7.78	-4.29	-9.89	-16.59	-12.49	-9.44	-13.92	-7.97	-3.77
	Interact Surface	582.2	718.1	559.8	824.1	596.3	977.7	819.5	633.3	594.3
	Intermolecular Energy (kcal/mol)	-9.74	-4.15	-9.06	-17.85	-14.13	-11.47	-15.94	-9.6	-2.61

**Quercetin**	Free energy (kcal/mol)	-5.6	-3.76	-7.46	-7.77	-7.18	-6.08	-8.82	-7.40	-6.28
	Interact Surface	562.5	602.1	613.0	662.4	630.2	520.6	734.0	644.6	624.4
	Intermolecular Energy (kcal/mol)	-5.9	-4.06	-7.76	-8.07	-7.48	-6.38	-9.12	-7.70	-6.58

**P-1:** DPP4; **P-2:** glutamine--fructose-6-phosphate transaminase; **P-3:** AMP-activated protein kinase; **P-4:** GLUT 2; **P-5:** SCD1**; P-6:** GLUT 4; **P-7:** sulfonylurea receptor; **P-8:** 11-beta hydroxysteroid dehydrogenase type 1; **P-9:** sodium/potassium/calcium exchanger.

## Data Availability

The data used to support the findings of this study are available from the corresponding author upon request.
